# The repeat length of *C9orf72* is associated with the survival of amyotrophic lateral sclerosis patients without *C9orf72* pathological expansions

**DOI:** 10.3389/fneur.2022.939775

**Published:** 2022-08-03

**Authors:** Lu Tang, Lu Chen, Xiaolu Liu, Ji He, Yan Ma, Nan Zhang, Dongsheng Fan

**Affiliations:** ^1^Department of Neurology, Peking University Third Hospital, Beijing, China; ^2^Beijing Key Laboratory of Biomarker and Translational Research in Neurodegenerative Diseases, Peking University Third Hospital, Beijing, China; ^3^Key Laboratory for Neuroscience, National Health Commission/Ministry of Education, Peking University, Beijing, China

**Keywords:** amyotrophic lateral sclerosis, *C9orf72*, repeats length, survival, *ATXN2*

## Abstract

**Objective:**

To explore whether the repeat lengths of the chromosome 9 open reading frame 72 (*C9orf72*) gene and the ataxin-2 (*ATXN2*) gene in amyotrophic lateral sclerosis (ALS) patients without *C9orf72* repeat expansions confer a risk of ALS or survival disadvantages in ALS.

**Methods:**

We screened a hospital-based cohort of Chinese patients with sporadic ALS without *C9orf72* repeat expansions and neurologically healthy controls for *C9orf72* GGGGCC and *AXTN2* CAG repeat length to compare the frequency of possible detrimental length alleles using several thresholds. Furthermore, the clinical features of ALS were compared between patients with ALS subgroups using different length thresholds of maximum *C9orf72* and *ATXN2* repeat alleles, such as sex, age of onset, diagnostic delay, and survival.

**Results:**

Overall, 879 sporadic patients with ALS and 535 controls were included and the repeat lengths of the *C9orf72* and *ATXN2* were both detected. We found significant survival differences in patients using a series of *C9orf72* repeat length thresholds from 2 to 5, among which the most significant difference was at the cutoff value of 2 (repeats 2 vs. >2: median survival 67 vs. 55 months, log-rank *p* = 0.032). Furthermore, Cox regression analysis revealed the role of age of onset [hazard ratio (HR) 1.04, 95% CI 1.03–1.05, *p* < 0.001], diagnostic delay (0.95, 0.94–0.96, *p* < 0.001), and carrying *C9orf72* repeat length of 2 (0.72, 0.59–0.89, *p* = 0.002) in the survival of patients without *C9orf72* repeat expansions. In addition, bulbar onset was associated with poorer survival when the patients carried the maximum *C9orf72* repeat allele over 2 (1.81, 1.32–2.48, *p* < 0.001). However, no survival difference was found when applying a series of continuous cutoff values of *ATXN2* or stratified by *C9orf72* repeats of 2.

**Conclusion:**

The length of 2 in the maximum *C9orf72* repeat allele was identified to be associated with favorable survival in ALS patients without *C9orf72* repeat expansions. Our findings from the clinical setting implicated the possible cutoff definition of detrimental C9orf72 repeats, which should be helpful in the understanding of genetics in ALS and in clinical genetic counseling.

## Introduction

Amyotrophic lateral sclerosis (ALS) is a fatal and rare neurodegenerative disorder characterized by involvement of the motor system and a final lethal course within 2–4 years after symptom onset (1). Since Superoxide Dismutase 1 (*SOD1*) mutations were identified to cause ALS in 1993 (2), a number of causative genes of ALS have been found. A large hexanucleotide (GGGGCC) repeat expansion (HRE) in the chromosome 9 open reading frame 72 (*C9orf72*) gene has been identified as the most frequent genetic cause in familial and sporadic patients with ALS and frontotemporal dementia (FTD) in Caucasian populations (3, 4), strongly arguing for a central role of *C9orf72* in ALS pathogenesis. However, pathological HRE is rarely observed in familial and sporadic ALS in Asia (5–8). HREs are usually hundreds to thousands of repeats in length. Older age of onset and bulbar onset were associated with shorter survival of patients with *C9orf72* HRE (9). On the other hand, there has not been a wide consensus on the minimum pathogenic repeat length, since the threshold of 30 repeats has been commonly used. Further studies indicated that the intermediate-length allele of *C9orf72* under 30 repeats might also be a pathological risk factor for ALS (10, 11) and other neurodegenerative disorders (12), such as Huntington's disease phenocopies, Parkinsonism, and schizophrenia. The varying thresholds have been justified in many mechanisms, e.g., effects on DNA methylation (repeats 7–24) (13) and gene expression (repeats 17–29) (14), and association with neurodegenerative disorders [repeats 17–30 (14), 20–22 (15)].

Previous studies have suggested that when genetically interacting with another major modifier of ALS, ataxin-2 (*ATXN2*), *C9orf72* depletion caused by pathologically expanded HRE resulted in the ALS-FTD pathogenesis (16). Intermediate *ATXN2* repeats may render *C9orf72* HRE carriers more susceptible to the development of ALS (17). The intermediate CAG repeats in *ATXN2* (size between 24 and 33) have been revealed to present a significant association with ALS as a relatively common ALS disease susceptibility gene (18). Since that report, several studies have reported similar results but with different cutoff values of repeat length for the risk (19). However, the possible interaction between non-pathological *C9orf72* repeats and *ATXN2* repeats in the clinical setting has been implicated but not been fully investigated.

To date and to our knowledge, there are limited data on the prognostic effect of the non-pathological *C9orf72* repeats on patients with ALS, which may have more implications for the Chinese ALS population, since there are a few patients with pathological *C9orf72* HRE in China. The survival and prognosis within a specific genetic context are important to our understanding of the genetics and pathophysiology of ALS. Here, we examined whether a *C9orf72* repeat length under 30, commonly reported clinical and demographic variables, and additional genetic variants [*ATXN2* intermediate repeats, *SOD1*, fused in sarcoma (*FUS*), and TAR DNA-binding protein (*TARDBP*)] are associated with survival in ALS patients with non-pathological *C9orf72* repeats.

## Methods

### Subjects

The individuals in this study were recruited at a national referral motor neuron disease clinic at the Department of Neurology, Peking University Third Hospital (PUTH), Beijing, between 2005 and 2012. The patients were examined and diagnosed with definite, probable, or possible ALS according to Airlie House diagnostic criteria (20) by specialist neurologists. Demographic information included sex, date of birth, and month/year of disease onset. Clinical data included the site of onset, first clinical symptom, date of diagnosis, date of the last follow-up, month/year of death, or invasive ventilation (if applicable, both were defined as end point events). Demographic and clinical features were collected during the first visit to the hospital and updated *via* telephone follow-ups every 3 months. Individuals without neurological disease history were used as controls of Chinese origin.

All participants provided written informed consent to participate in the genotype-phenotype studies approved by the institutional ethics committee of PUTH.

### Genetic analysis

Genomic DNA was extracted from peripheral blood using standard protocols. Two-step PCR (fluorescent fragment-length analysis and then repeat-primed PCR) was performed to assess the length of *C9orf72* repeat alleles as previously reported (6). The CAG repeat size in *ATXN2* was determined by PCR and subsequent fluorescent fragment-length analysis on an ABI 3730XL genetic analyzer as described previously (19). Some of the subjects had previously been screened for *C9orf72* (7) and *ATXN2* (19), and the results for these two genes were published separately. Patients carrying pathological *C9orf72* HRE (repeat size ≥ 30) were excluded from this study. Among the patients, subjects with adequate DNA samples were detected by Sanger sequencing for all exons in the frequent ALS-causing genes *SOD1*, fused in sarcoma (*FUS*), and TAR DNA-binding protein (*TARDBP*; indicated as *SoFT* mutation status below and *SoFT* was abbreviated for the *SOD1, FUS*, and *TARDBP* genes).

### Statistics

Due to the exploratory nature of this study, we defined the larger number of both *C9orf72* and *ATXN2* repeats as the maximum repeat length of alleles. The differences in the maximum repeat length of *C9orf72* (defined as MaxC9) and the maximum repeat length of *ATXN2* (MaxATXN2) were compared between patients with ALS and neurologically healthy controls to assess whether they could be risk factors for ALS by Mann-Whitney U tests (continuous variable) and Pearson's chi-squared test or two-tailed Fisher's exact tests (categorical variable using several repeat length cutoff values), respectively.

To further investigate the role of non-pathological length repeats of *C9orf72* and *ATXN2* in survival and prognosis, different cutoff values of MaxC9 and MaxATXN2 were used to categorize the patients into subtypes. For these extra analyses, Student's t*-*test and Mann-Whitney U tests were then used to analyze the difference in the age of onset and the diagnostic delay from symptom onset (the interval from the symptom onset to the diagnosis), respectively. Kaplan-Meier analysis was performed using different cutoff values of MaxC9 and MaxATXN2 to obtain the survival curves, and survival differences between patient subgroups were determined by a log-rank test. Additionally, we utilized Cox proportional hazards regression models for sex, age of onset, diagnostic delay, site of onset, *C9orf72, ATXN2*, and *SoFT* mutation status to address the potential confounding influence of these variables on the survival of ALS patients without *C9orf72* HRE.

A two-tailed *p* < 0.05 was considered statistically significant. All analyses were performed and visualized using R Statistical Software (version 3.6.3).

## Results

### Demographic and clinical features

A total of 879 patients diagnosed with sporadic ALS and 535 neurologically normal controls were included and analyzed in the present study. The demographic descriptions of the patients and controls are summarized in Table 1. There were 547 men among the patients with ALS (62.2%) and 328 men among the controls (61.3%). The mean age of onset of patients with ALS was 50.6 years old (95% Cl 49.8–51.5). There was no significant difference in the age of DNA sampling between the patients with ALS and the controls (51.4 vs. 51.8 years, *p* = 0.54). Among 748 patients with available data, 120 patients (16%) had the bulbar onset.

**Table 1:** Demographic features of patients ALS and controls.

	**ALS patients**	**Controls**	***p* value**
Total	879	535	
Male (*n*, %)	547 (62.2%)	328 (61.3%)	0.74
Age of onset, years (mean, 95% CI)	50.6 (49.8–51.5)	NA	
Age of sampling (mean, 95% CI)	51.4 (50.5–52.2)	51.8 (50.7–52.9)	0.54
Bulbar onset (*n*, %)	120 (16.0%)^#^	NA	

# *There are 748 patients with available data of onset site*.

### Genetic findings and ALS risk analysis of *C9orf72* and *ATXN2* repeat length

**Figure 1:**
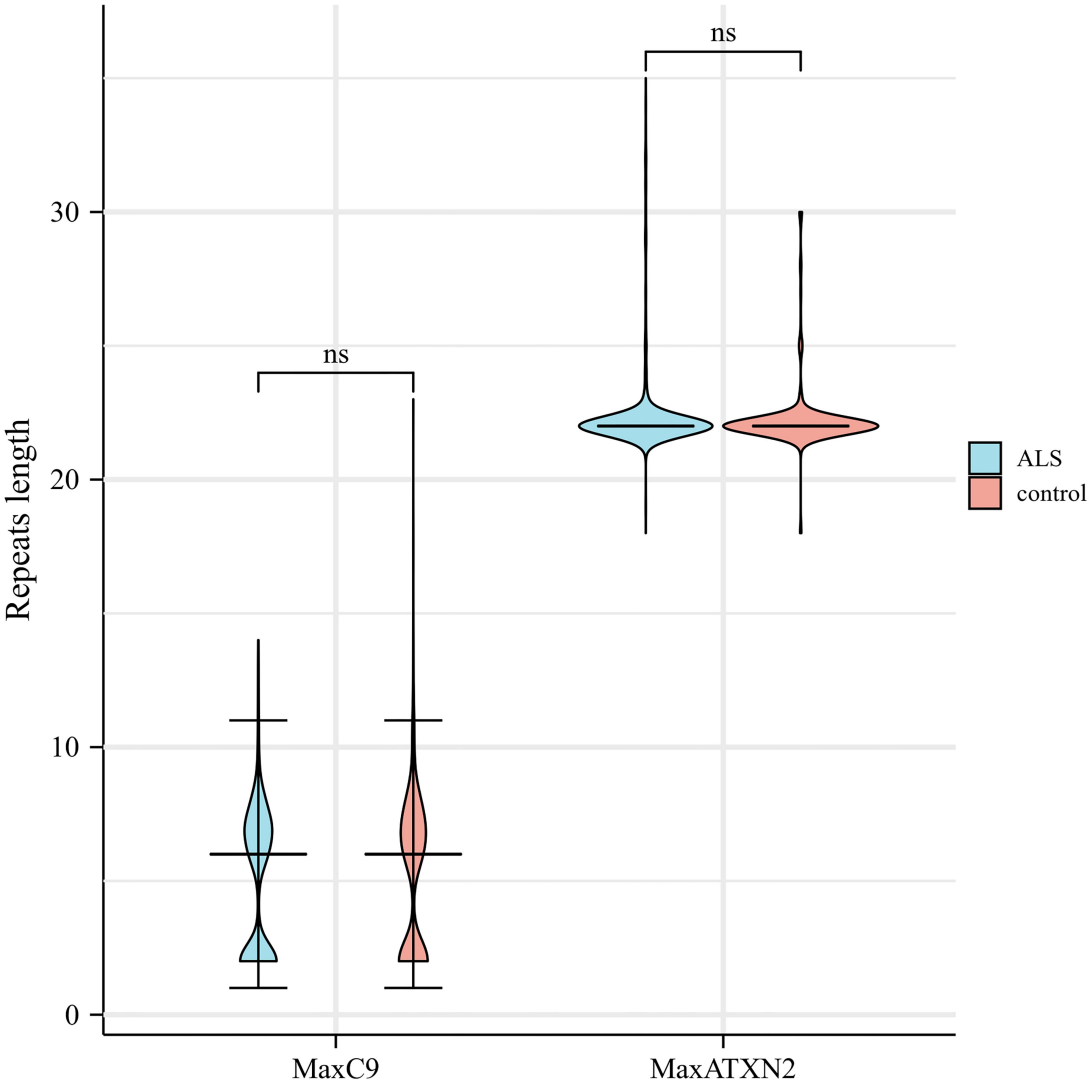
Distribution of C9orf72 GGGGCC and ATXN2 CAG repeat length in patients with ALS and controls. ns, not significant.

**Figure 2:**
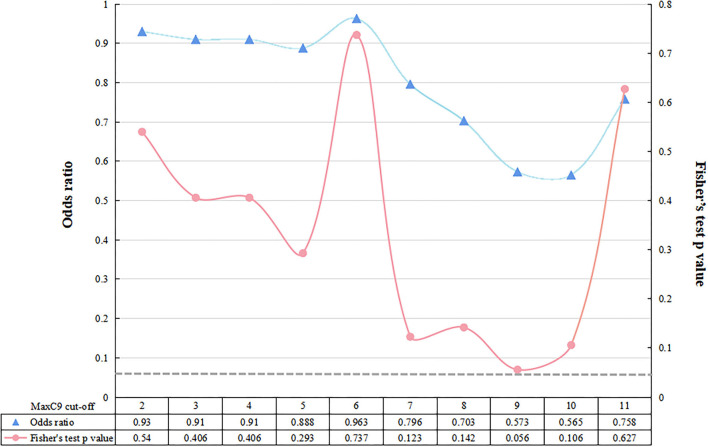
Fisher's exact test between patients with ALS and controls at each cutoff value of *C9orf72* repeat. The dotted gray line represents the value of *p* < 0.05 at a significance level.

The median [interquartile rage (IQR)] repeat length of MaxC9 in 879 patients with ALS was 6 (2–7), whereas that in controls was identical to 6 (2–7) (Mann-Whitney U test, *p* = 0.302, Figure 1). Using a series of *C9orf72* repeat length cutoff values, no significant difference in frequency between patients with ALS and controls was identified (Fisher's exact test at each cutoff value, all *p* > 0.05, Figure 2). The median (range) repeat length of MaxATXN2 in patients with ALS was 22 (18–35) while it was 22 (18–30) in controls, with 22 repeats being the most frequent in both patients with ALS (91.4%) and controls (92.7%, Figure 1). When using an *ATXN2* repeat length cutoff value ≥31, the difference in frequency between patients with ALS and controls was statistically significant (Pearson's chi-squared test, *p* = 0.003, Supplementary Table S1).

Among the patients with ALS, 537 patients with enough DNA samples were screened for the *SoFT* mutation status, identifying 8 patients with *SOD1* mutations, 19 with *FUS* mutations, and 9 with *TARDBP* mutations.

### Survival and phenotype analysis of patients without *C9orf72* HRE using a series of repeat length thresholds

Among the 879 patients with ALS, there were 726 patients with detailed survival time and therefore they were included for the further survival analysis. There were 349 cases with censored data and 377 with end point events, showing no significant difference in the *MaxC9* distribution of by Mann-Whitney U test (*p* = 0.09).

**Figure 3:**
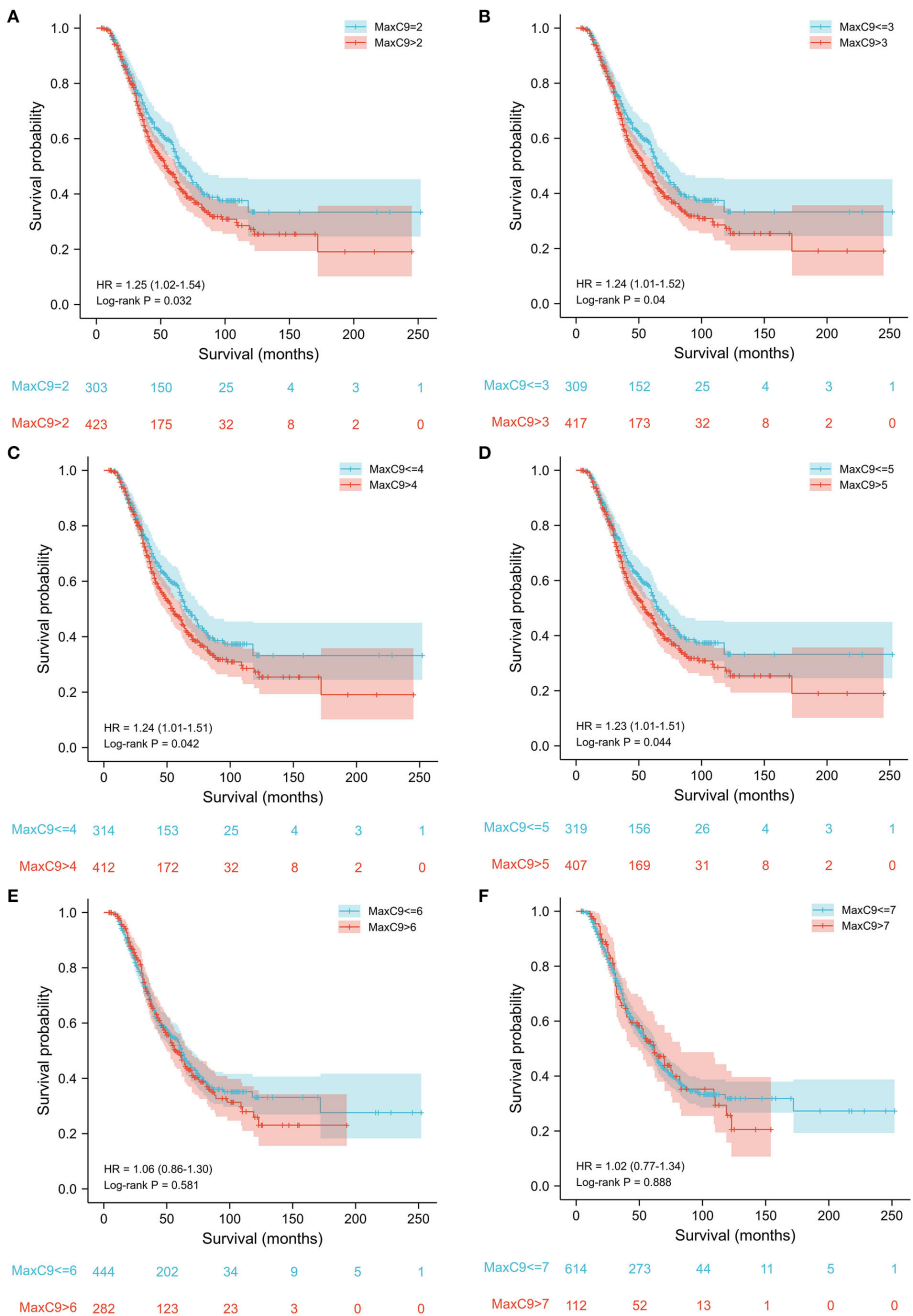
Survival analysis of patients without *C9orf72* HRE using a series of cutoff values of GGGGCC repeat length from 2 to 7. Cutoff values of 2–5 **(A–D)** could discriminate the survival difference, while cutoff values of 6 and 7 **(E,F)** could not.

A series of cutoff values from 2 to 5 of MaxC9 discriminated patients in survival time with statistical significance (all *p* < 0.05), most significant at repeat length 2 (repeats 2 vs. >2: median 67 months vs. 55 months, *p* = 0.032, Figures 3A–D). With a cutoff of 6 and more, the difference in survival was no longer significant ( ≤ 6 vs. >6: 62 vs. 57, *p* = 0.58; ≤ 7 vs. >7: 62 vs. 61, *p* = 0.89, Figures 3E,F). To explore the further differences in survival and phenotypes within the context of detrimental *C9orf72* repeats, we applied the most significant cutoff value of 2, which could include 41.7% of patients in the present study, to determine the potential effect in the following comparisons. Meanwhile, no survival difference was found when applying a series of continuous cutoff values of MaxATXN2 or stratifying by MaxC9 of 2 (Supplementary Table S2).

In total, there was no survival difference between male patients and female patients (median 58 vs. 66 months, log-rank *p* = 0.145). When stratified by *C9orf72* repeat length of 2, males with length over 2 (*n* = 281) showed survival disadvantage, when compared with males with length of 2 (*n* = 186; median 49 vs 65 months, *p* = 0.033); females over 2 (*n* = 142) did not present significantly shorter survival than those with 2 (*n* = 117; 64 vs 72 months, *p* = 0.57).

Taking the known *SoFT* mutation status in 537 patients into comparison, the marginally significant difference in survival time was present in patients with *TARDBP* mutations [*TARDBP*(+): 2 vs. >2: 58.1 vs. 16.3 months, *p* = 0.048], while there was no significant difference in patients carrying *SOD1* (log-rank *p* = 0.22), *FUS* (log-rank *p* = 0.55), or SoFT(–) (log-rank *p* = 0.08).

Univariate Cox regression analysis (Table 2) showed that a poorer prognosis was observed with an older age of onset (*p* < 0.001), bulbar site of onset (*p* < 0.001), and carrying *TARDBP* mutation (*p* = 0.024), while a longer survival was observed with a longer diagnostic delay (*p* < 0.001) or carrying *C9orf72* repeat length of 2 (*p* = 0.032). Further multivariate analysis (Table 2) confirmed the role of age of onset [hazard ratio (HR) 1.04, 95% CI 1.03–1.05, *p* < 0.001], diagnostic delay (0.95, 0.94–0.96, *p* < 0.001) and carrying *C9orf72* repeat length of 2 (0.72, 0.59–0.89, *p* = 0.002) in the survival of patients without *C9orf72* HRE.

**Table 2:** Cox regression analysis of patients without *C9orf72* expansions.

**Characteristics**	**Total (*N*)**	**Univariate analysis**		**Multivariate analysis**	
		**Hazard ratio (95% CI)**	***p* value**	**Hazard ratio (95% CI)**	***p* value**
Sex	726		0.137		
Male	467	Reference			
Female	259	0.85 (0.69–1.05)	0.137		
Age of onset (years)	726	1.04 (1.03–1.05)	**<0.001**	1.04 (1.03–1.05)	**<0.001**
Diagnostic delay (months)	723	0.95 (0.94–0.96)	**<0.001**	0.95 (0.94–0.96)	**<0.001**
Site of onset	717		**<0.001**		
Spinal	604	Reference			
Bulbar	113	1.58 (1.22–2.05)	**<0.001**	1.02 (0.78–1.33)	0.89
*ATXN2*	726	0.96 (0.89–1.03)	0.218		
*SoFT* Status	537		0.105		
*SoFT* (–)	501	Reference			
*SOD1* (+)	8	0.89 (0.28–2.79)	0.839		
*FUS* (+)	19	1.41 (0.75–2.65)	0.290		
*TARDBP* (+)	9	2.55 (1.13–5.73)	**0.024**		
*C9orf72*	726				
*C9orf72* > 2	423	Reference			
*C9orf72* = 2	303	0.80 (0.65–0.98)	**0.032**	0.72 (0.58–0.89)	**0.002**

*The bold values indicate statistically significant values of p < 0.05*.

**Figure 4:**
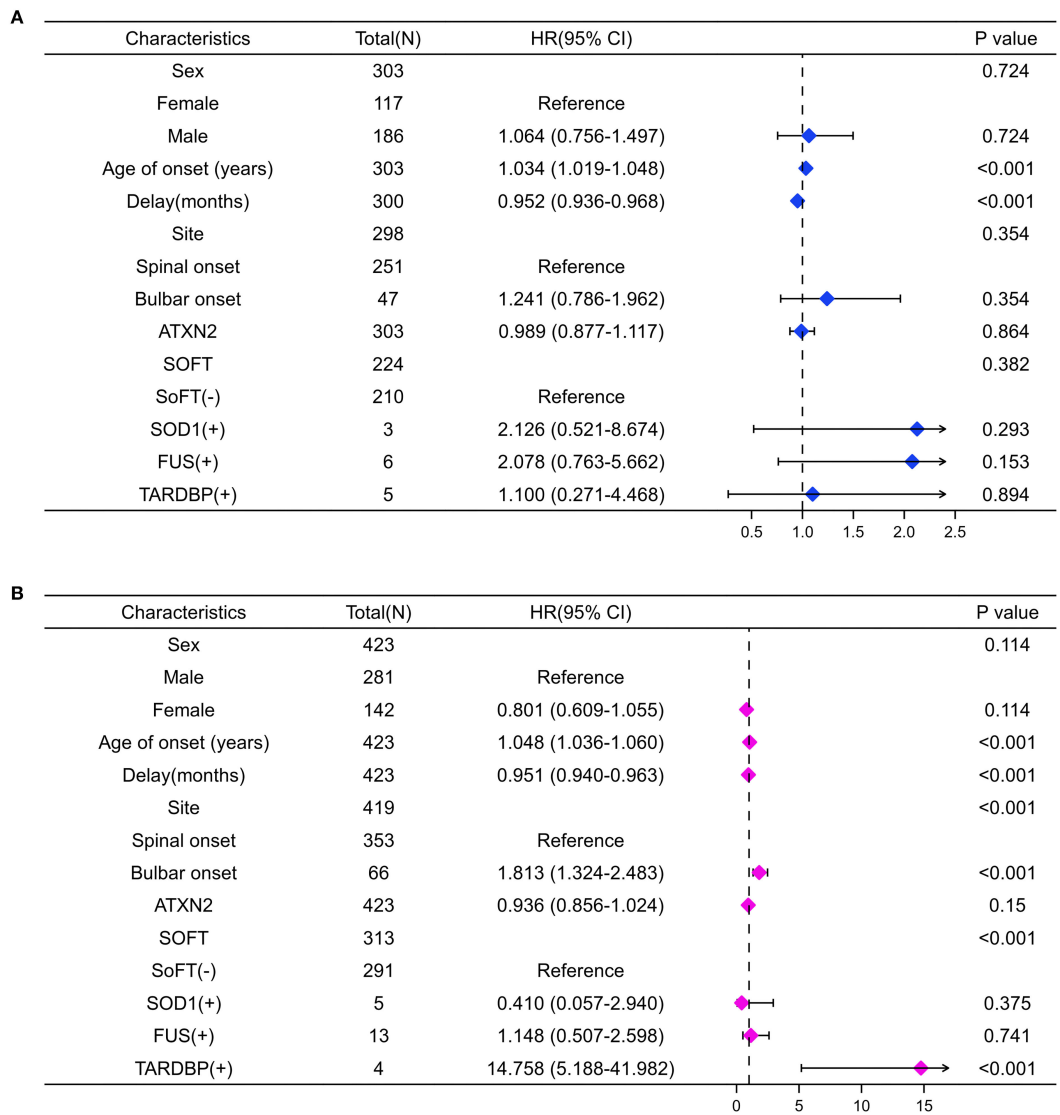
Forest plots summarize the results of univariate Cox analysis stratifying by *C9orf72* repeat length. When MaxC9 = 2 **(A)**, the age of onset and the delay are associated with the survival of patients (both *p* < 0.001). When MaxC9 > 2 **(B)**, besides the age of onset and the delay, the bulbar site of onset and carrying *TARDBP* mutation are also associated with the survival of patients (all *p* < 0.001).

We further performed the univariate analysis stratified by *C9orf72* repeat length of 2. Interestingly, the forest plots in Figure 4 indicate that bulbar onset (HR 1.81, 95% CI 1.32–2.48, *p* < 0.001) or carrying *TARDBP* mutations (14.76, 5.19–41.98, *p* < 0.001) could confer a survival disadvantage only when the *C9orf72* repeat length was over 2.

Additionally, we applied a series of continuous cutoff values of *C9orf72* and *ATXN2* to subdivide the patients to determine the possible association with clinical features of the age of onset and diagnostic delay. However, no significant differences were found in either clinical phenotype using different repeat length thresholds of *C9orf72* and *ATXN2* (Supplementary Tables S3, S4).

## Discussion

In the light of the shared features of intermediate length repeats and the pathological interaction of *C9orf72* and *ATXN2* genes in ALS pathogenesis, we performed the joint analyses of non-pathological repeats of *C9orf72* (i.e., < 30) and intermediate repeats of *ATXN2* (18–35) in 879 patients with ALS and 535 neurologically healthy controls, aiming to explore the risk of ALS and prognostic factors in ALS patients without *C9orf72* HRE. Although we did not find any association of intermediate *ATXN2* length with survival or clinical interaction with *C9orf72*, our results revealed that carrying *C9orf72* repeats of 2 was associated with longer survival, which has not been identified in previous studies. Furthermore, Cox regression analysis confirmed the protective role of the younger age of onset, longer diagnostic delay, and carrying *C9orf72* repeat length of 2 in the survival of patients without *C9orf72* HRE.

The HRE within the first intron of the *C9orf72* gene has been the most frequent genetic cause of ALS and FTD worldwide (21). Major efforts have been dedicated to reveal the mechanism of toxicity through which *C9orf72* HRE leads to neurodegeneration. Pathological HRE was shown to affect *C9orf72* expression with reduced transcript and protein levels consistently measured in pathological tissues from patients with ALS and FTD (14). It is suggested that the intermediate *C9orf72* alleles are “predisposing” to lead to large expansions over subsequent generations and affect the normal transcriptional activity of the C9orf72 promoter. The threshold for pathogenicity is usually defined as 30 mainly due to technical limitations of the repeat-primed PCR technique. The possible pathological role of intermediate length GGGGCC between 20 and 30 has also been multiply suggested (13, 22), without a generally accepted cutoff value. In addition, 20–22 repeats were reported to be pathogenic in patients with FTD (15). We have not identified with any cutoff value that the non-pathological length (<30) of *C9orf72* repeats was associated with the risk of ALS. This may result from the lack of alleles with repeats over 20 in ALS cases in our cohort. The risk of short repeats (<20) might be too minor to be detected in a relatively small-size case-control study.

Previous literature has suggested that *C9orf72* HRE carriers have an unfavorable prognosis in patients with ALS (9). However, a few studies have investigated the association between the repeat length of *C9orf72* and the survival of ALS patients without *C9orf72* HRE. In our analysis, the survival difference resulting from a series of repeat length cutoff values from 2 to 5 expanded our understanding of the potential detrimental repeat length for patients with ALS. More clinical phenotype-genotype studies are needed to clarify the prognostic role of repeat lengths over 5, which usually represent more than 50% of sporadic ALS patients without *C9orf72* HRE in Asia [this study, 56%; another study in China, 64% (23); and Korea, 50.3% (24)]. In addition, it should be kept in mind that while *C9orf72* HRE is known to decrease *C9orf72* expression, intermediate *C9orf72* repeats resulted in increased *C9orf72* expression in both human brain tissue and CRISPR/cas9 knockin iPSC-derived neural progenitor cells (14). Therefore, with the upcoming gene-modifying therapies targeting *C9orf72*, the strategy for HRE and intermediate or short repeats should be carefully designed with caution.

The multivariate Cox regression model used in the present study showed that the predictors of favorable survival were younger age of onset, longer diagnostic delay, and carrying *C9orf72* repeats of 2. However, the sex effect was not identified here, which has been regarded as a prognostic predictor in previous studies (1). *C9orf72* HRE is not fully penetrant until the age of 80 years (21). Additionally, *C9orf72* depletion in neuronal cultures led to the accumulation of unresolved aggregates of SQSTM1/p62 involved in macroautophagy/autophagy and phosphorylated TDP-43 (16). *C9orf72* reduction alone, however, did not trigger major neuronal toxicity, suggesting that extra stress is probably required to induce neuronal death upon *C9orf72* loss of function. We, therefore, hypothesized that additional pathological mutations may affect the survival of patients carrying *C9orf72* repeats of 2 and our finding (4 patients with repeats >2 vs. 5 patients = 2, 16.3 vs. 58.1 months, *p* = 0.048) was in line with both our hypothesis and previous studies of *C9orf72* and *TARDBP* (25, 26) *in vitro*, although more clinical evidence is required due to the small number of patients with *TARDBP* mutations. The role of *C9orf72* in ALS pathophysiology involves the TDP-43 inclusion function. It is reasonable that the mutation of the *TARDBP* gene exaggerates the potential toxicity of the *C9orf72* HRE. Conversely, a similar synergistic effect was not observed in patients carrying *SOD1* or *FUS* mutations in our study, supporting that *C9orf72* loss of function did not accentuate the toxicity of *FUS* and *SOD1* mutations in neuronal culture (16).

To date, hands of studies have focused on the effect of intermediate length *ATXN2* on *C9orf72* pathogenicity. Partial *C9orf72* depletion coupled with intermediate repeats (30×), but not normal-size (22×), of *ATXN2* was associated with *ATXN2* aggregation and neuronal cell death confirmed in both *in vitro* and *in vivo* (16). *ATXN2* intermediate length repeats have been identified as a risk factor for ALS (18, 27) through modifying TDP-43 toxicity (18) and a predictor of reduced survival in patients with ALS (28). Indeed, intermediate *ATXN2* repeats are also a risk factor for ALS patients with *C9orf72* HRE (17) and a possible modifier of disease phenotypes (clinical type and age of onset) of HRE carriers (29) with conflicting evidence. In the present study, we compared the *ATXN2* repeat length between patients with ALS and controls. Consistent with our published report (19), the cutoff of 31 repeats in *ATXN2* (MaxATXN2) has shown a significant risk association with patients with ALS. However, the length of *ATXN2* repeats did not show any association with the age of onset, diagnostic delay, or survival time in the present study, even when stratified by the *C9orf72* repeat length of 2. Due to the potential discrepant pathogenesis between *C9orf72* HRE with reduced expression and the intermediate length of *C9orf72* with increased expression, the suggested role of *ATXN2* in *C9orf72* HRE carriers should be re-evaluated in ALS patients without *C9orf72* HRE.

Limitations of our study were that our patients and controls were all of Chinese origin, and it was not a large cohort regarding low prevalence but high complexity of ALS and potential minor effect of *C9orf72* and *ATXN2* genes. Additionally, we have no data about the cognitive and psychiatric status of the patients included. It hereby was deficient to explore the association between these data and different *C9orf72*-related manifestations, especially cognitive and psychiatric symptoms.

Our study identified that non-pathological *C9orf72* repeat length was associated with the survival of ALS patients without *C9orf72* HRE, which was especially meaningful for patients with ALS in Asia when the pathological *C9orf72* HRE was quite rare when compared with Caucasian patients. Our findings add confirmatory evidence on the prognostic significance of the older age and bulbar onset, diagnostic delay, and a suggested modifying role of *TARDBP* mutations in *C9orf72*-related ALS. The cutoff value of *C9orf72* repeats, however, could vary in different populations and cohorts. Further association studies between the length of *C9orf72* repeats and survival and other phenotypes are needed.

## Data availability statement

The datasets presented in this study can be found in online repositories. The names of the repository/repositories and accession number(s) can be found in the article/Supplementary material.

## Ethics statement

The studies involving human participants were reviewed and approved by Institutional Ethics Committee of Peking University Third Hospital. The patients/participants provided their written informed consent to participate in this study.

## Author contributions

LT and DF conceived and designed the study and analyzed and interpreted the data. LT, LC, YM, and NZ contributed major role in the acquisition of clinical data. LT, XL, and JH contributed major role in the acquisition of genetic results. LT, LC, and DF drafted the manuscript of intellectual content and revised the manuscript for intellectual content. All authors contributed to the article and approved the submitted version.

## Funding

This study was funded by the National Natural Science Foundation of China (81873784 and 82071426 to DF and 81901298 to LT) and the Clinical Cohort Construction Program of Peking University Third Hospital (BYSYDL2019002 to DF).

## Conflict of interest

The authors declare that the research was conducted in the absence of any commercial or financial relationships that could be construed as a potential conflict of interest.

## Publisher's note

All claims expressed in this article are solely those of the authors and do not necessarily represent those of their affiliated organizations, or those of the publisher, the editors and the reviewers. Any product that may be evaluated in this article, or claim that may be made by its manufacturer, is not guaranteed or endorsed by the publisher.
