# Supplementary material for: The repeat length of C9orf72 is associated with the survival of amyotrophic lateral sclerosis patients without C9orf72 pathological expansions
Source: Front Neurol. 2022 Aug 3;13:939775. doi: 10.3389/fneur.2022.939775 (PMC9381700; doi:10.3389/fneur.2022.939775)
Supplement: Supplementary file 1 [file Data_Sheet_1.pdf]

Supplementary Table 1. Fisher's exact test between ALS cases and controls at each MaxAXTN2 cut-off value

	22 vs >22	≤23 vs >23	≤24 vs >24	≤25 vs >25	≤26 vs >26	≤27 vs >27	≤28 vs >28	≤29 vs >29	≤30 vs >31
ALS	808 vs 71	830 vs 49	839 vs 40	848 vs 31	851 vs 28	854 vs 25	856 vs 23	862 vs 17	865 vs 14
Control	500 vs 35	509 vs 26	509 vs 26	520 vs 15	521 vs 14	523 vs 12	527 vs 8	527 vs 8	535 vs 0
P value	0.30	0.63	0.80	0.54	0.63	0.61	0.19	0.53	0.003*

* Pearson *Chi*-square test.

Supplementary Table 2. Association of *ATXN2* repeat length (MaxATXN2) with survival by log-rank test

	**22 vs >22**	**≤23 vs >23**	**≤24 vs >24**	**≤25 vs >25**	**≤26 vs >26**	**≤27 vs >27**	**≤28 vs >28**	**≤29 vs >29**	**≤30 vs >31**
**Median, months**	61 vs 64	61 vs 75	61 vs 55	61 vs 54	61 vs 75	61 vs 44	61 vs 75	61 vs NA	61 vs NA
**P value**	0.14	0.28	0.52	0.63	0.54	0.75	0.44	0.29	0.067
**MaxC9 >2 (n=423)**									
**Median, months**	53 vs 75	53 vs 75	53 vs 75	53 vs 75	55 vs 75	55 vs 75	55 vs 75	55 vs NA	53 vs NA
**P value**	0.08	0.17	0.26	0.29	0.42	0.42	0.42	0.28	0.08
**MaxC9 =2 (n=303)**									
**Median, months**	67 vs 54	67 vs 54	67 vs 43	67 vs 43	67 vs 43	67 vs 37	65 vs 43	65 vs 43	65 vs NA
**P value**	0.86	0.95	0.70	0.58	0.96	0.61	0.68	0.68	0.40

724 patients' survival data available

Supplementary Table 3. Association of *C9orf72* repeat length (MaxC9) and *ATXN2* repeat length ((MaxATXN2) with age of onset by *t* test

**MaxC9**									
	2 vs >2	≤3 vs >3	≤4 vs >4	≤5 vs >5	≤6 vs >6	≤7 vs >7	≤8 vs >8	≤9 vs >9	≤10 vs >10
**Mean, years**	50.2 vs 50.9	50.3 vs 50.9	50.3 vs 50.9	50.3 vs 50.9	50.4 vs 51.0	50.4 vs 52.2	50.6 vs 52.1	50.6 vs 52.6	50.6 vs 51.8
**P value**	0.40	0.41	0.41	0.45	0.45	0.12	0.45	0.43	0.71
**MaxATXN2**									
	22 vs >22	≤23 vs >23	≤24 vs >24	≤25 vs >25	≤26 vs >26	≤27 vs >27	≤28 vs >28	≤29 vs >29	≤30 vs >31
**Mean, years**	50.6 vs 50.7	50.6 vs 51.6	50.6 vs 51.8	50.6 vs 50.8	50.6 vs 50.6	50.6 vs 51.1	50.7 vs 49.9	50.6 vs 51.2	50.7 vs 49.4
**P value**	0.98	0.58	0.54	0.93	0.98	0.86	0.76	0.84	0.71

Supplementary Table 4. Association of *C9orf72* repeat length (MaxC9) and *ATXN2* repeat length (MaxATXN2) with diagnostic delay by Mann-Whitney test

**MaxC9**									
	2 vs >2	≤3 vs >3	≤4 vs >4	≤5 vs >5	≤6 vs >6	≤7 vs >7	≤8 vs >8	≤9 vs >9	≤10 vs >10
**P value**	0.44	0.54	0.54	0.64	0.71	0.69	0.43	0.45	0.74
**MaxATXN2**									
	22 vs >22	≤23 vs >23	≤24 vs >24	≤25 vs >25	≤26 vs >26	≤27 vs >27	≤28 vs >28	≤29 vs >29	≤30 vs >31
**P value**	0.73	0.22	0.50	0.77	0.83	0.70	0.64	0.85	0.89
